# Genome-wide microarray analysis leads to identification of genes in response to herbicide, metribuzin in wheat leaves

**DOI:** 10.1371/journal.pone.0189639

**Published:** 2017-12-11

**Authors:** Whitney Pilcher, Hana Zandkamiri, Kelly Arceneaux, Stephen Harrison, Niranjan Baisakh

**Affiliations:** School of Plant, Environmental, and Soil Sciences, Louisiana State University Agricultural Center, Baton Rouge, Louisiana, United States of America; Estacion Experimental del Zaidin, SPAIN

## Abstract

Herbicides are an important component of weed management in wheat, particularly in the southeastern US where weeds actively compete with wheat throughout the winter for nutrients and reduce tillering and ultimately the yield of the crop. Some wheat varieties are sensitive to metribuzin, a low-cost non-selective herbicide, leading to leaf chlorosis, stand loss, and decreased yield. Knowledge of the genetics of herbicide tolerance in wheat is very limited and most new varieties have not been screened for metribuzin tolerance. The identification of genes associated with metribuzin tolerance will lead to the development of molecular markers for use in screening breeding lines for metribuzin tolerance. AGS 2035 and AGS 2060 were identified as resistant and sensitive to metribuzin in several previous field screening experiments as well as controlled condition screening of nine varieties in the present study. Genome-wide transcriptome profiling of the genes in AGS 2035 and AGS 2060 through microarray analysis identified 169 and 127 genes to be significantly (2-fold, P>0.01) up- and down-regulated, respectively in response to metribuzin. Functional annotation revealed that genes involved in cell wall biosynthesis, photosynthesis and sucrose metabolism were highly responsive to metribuzin application. (Semi)quantitative RT-PCR of seven selected differentially expressed genes (DEGs) indicated that a gene coding for alkaline alpha-galactosidase 2 (AAG2) was specifically expressed in resistant varieties only after one and two weeks of metribuzin application. Integration of the DEGs into our ongoing mapping effort and identification of the genes within the QTL region showing significant association with resistance in future will aid in development of functional markers for metribuzin resistance.

## Introduction

Weed control in wheat (*Triticum aestivum*) is an important aspect of crop management, particularly in the southeastern US where wheat and weeds actively grow throughout the winter. Weed pressure in seedling wheat negatively affects tillering and thereby reduces yield of the crop. Wild oats, in excess of 200 plants per square meter, can reduce tillering in wheat by more than 50% and yield by as much as 66% [1]. Use of herbicides is the most common management practice to control weed infestations in wheat. Some non-selective herbicides, such as metribuzin, are advantageous over selective herbicides because of the flexibility of their use as pre- and postemergent, and these are less labor-intensive with less or no tillage requirement, and most importantly cost-effectiveness as a single application can kill many types of weeds in the field. Despite their advantages, non-selective herbicide resistance has not been widely targeted in breeding field crops becasue they may not discriminate between the weed and the crop.

Metribuzin [4 4-Amino-6-tert-butyl-3-methylsulfanyl-1,2,4-triazin-5-one] is a non-selective herbicide that belongs to the chemical class of triazinones (also called triazines). Metribuzin hinders DNA synthesis in treated plants and acts on photosystem II, ultimately inhibiting photosynthesis [2]. Metribuzin is an important herbicide in wheat due to its broad spectrum of weed control and relatively low cost compared to other available wheat herbicides. Metribuzin provides good control of important annual grass and broad-leaf weeds including winter annual bluegrass (*Poa annua*), common chickweed (*Stellaria media*), henbit (*Lamium amplexicaule*), Italian ryegrass (*Lolium perenne* ssp. multiflorum) and corn buttercup (*Ranunculus arvensis*). Axiom^®^ is a newer wheat herbicide widely used as pre-plant or post-emergence weed control in wheat and is a mixture containing metribuzin.

Most wheat varieties show tolerance to metribuzin at the recommended usage rates of 225–300 ml/ha. However, some varieties are sensitive to metribuzin and can suffer stand reduction and yield loss. Reaction of wheat varieties to metribuzin is highly dependent on application rate and edaphoclimatic conditions, such as soil organic matter and texture, rainfall, and temperature. Metribuzin is highly soluble in water and excess rainfall can cause puddling resulting in injurious concentration of metribuzin in low spots in fields.

There have been numerous studies on reaction of wheat varieties to metribuzin. For example, Burgess et al. [3] screened 86 varieties at Mississippi State University for metribuzin resistance and classified 31 as tolerant, 9 as susceptible and 46 as moderately tolerant/susceptible. Metribuzin 75DF at 200 g/ha was shown to result in visible (15%) necrosis, which increased in intensity (25–35%) with the increase in metribuzin rates (400–600 g/ha) in all the varieties tested [4]. In addition, metribuzin at 400–600 g/ha caused 10–15% loss in biomass and significant yield loss when applied as pre-emergence. Metribuzin labels generally list tolerant and susceptible varieties of wheat. However, new wheat varieties are released each year and there are many currently grown wheat varieties that have not been fully characterized for metribuzin reaction. Field screening for metribuzin tolerance is costly and time-consuming, requiring application of higher than recommended levels of metribuzin in replicated yield trials over several environments.

Genetic control of tolerance to metribuzin has been investigated to a certain extent. However, information on the genetics of inheritance of metribuzin tolerance in wheat is very limited. Using two durum wheat (*Triticum durum*) varieties and their progenies, Villaroya et al. [5] showed a semidominance mode of inheritance of tolerance. The broad-sense and narrow-sense heritability of tolerance were 0.52 and 0.23, respectively. The inheritance of tolerance to metribuzin was complex and under the control of multiple alleles. This was supported by the observation that physiological processes, such as uptake, translocation and metabolism/detoxification modified the amount of herbicide reaching the target site [5]. On the other hand, Ratliff et al. [6], using as an assay with common wheat related to herbicide action at the target site, reported a nuclear as well as cytoplasmic inheritance of tolerance.

Use of wheat varieties resistant to metribuzin will be a viable solution for weed management by the producers. Conventional breeding takes about 8–12 years in wheat. Molecular marker-assisted breeding (MAB) can hasten the breeding cycle by 2–3 years. Therefore, identification of genes and quantitative trait loci (QTLs) that control the resistance to metribuzin is a prerequisite to development of linked molecular markers to facilitate marker-assisted selection (MAS). Here, we report on identification of candidate genes associated with the tolerance of wheat to metribuzin using the 44K wheat gene-chips.

## Materials and methods

### Plant materials and metribuzin treatment

Nine bread wheat varieties, AGS 2035, AGS 2060, Harrison, LA 754, LA 3200E2 (breeding line), Pio 26R87, Progeny 125, USG 3120 and USG 3555, known to have differential response to metribuzin under field condition [3], were seed-planted (two per pot) in 10.16 cm x 10.16 cm x 8.89 cm pots filled with potting soil consisting of 50% river silt and 50% organic potting mix. The pots were kept inside a growth chamber maintained at a 12 h light with a day/night temperature regime of 15/4°C. Seedlings at 4-leaf stage were sprayed with metribuzin (Sencor^®^ 4 DF herbicide, Bayer Inc) aqueous solution at 140 liters/ha. Observations were recorded on leaf chlorosis and burning after 1 week of herbicide application to determine the effect of herbicide. The experiment was conducted in three biological replications (two plants per replication).

### Measurement of photosynthetic yield

Maximum quantum yield of photosystem II, as a measure of damage to photosystem II, was recorded in dark adapted plants prior to and after 2 and 4 days of metribuzin application with a portable fluorometer (PAM-2100; Walz, Germany). The minimal fluorescence level (Fo) with all photosystem (PS) II reaction centers open was determined by measuring the modulated light, which was sufficiently low. Maximal fluorescence level (Fm) with all PSII reaction centers closed was determined by a 0.8-s saturating pulse in dark-adapted leaves. Maximum quantum yield of photosystem II was measured as Fv/Fm where Fv = Fm – Fo as described earlier [7].

### Tissue collection and microarray

Two varieties, AGS 2035 and AGS 2060 were chosen for the gene expression profiling based on the injury inflicted by metribuzin. Leaf tissues were collected at four different time points (24 h, 3 d, 7 d, and 14 d) from sprayed plants; unsprayed plants were used as control. Total RNA was isolated from the leaf tissues using RNeasy plant minikit (Qiagen, Valencia, CA). Qualitative and quantitative assessment of RNA was done using 1% fomamide gel and ND-1000 spectrophotometer (Nanodrop, Wilmington, DE).

In vitro amplification of RNA was performed using the Low-Input Quick Amp Labeling Kit (Agilent Technologies, Santa Clara, CA). The cRNA labeling, hybridization, washing and scanning were done using the protocol described by Baisakh et al. [8]. Briefly, the cRNAs were labeled with either Cy 5 or Cy 3 dye using same labeling kit. Dye-labeled cRNAs were purified using the Mini Prep Kit (Qiagen, Valencia, CA). Coupled dye-swapped cRNA samples were hybridized to wheat oligonucleotide microarrays (Agilent Technologies) for 17 hours at 60°C using the 2X Hi RPM Hyb Buffer of Gene Hybridization Kit (Agilent Technologies). The Agilent wheat microarrays (https://www.ncbi.nlm.nih.gov/geo/query/acc.cgi?acc=GPL13627) contained 43,815 gene sequences for 45,220 probes on a 4x44 microarray slide. After washing, the slides were scanned using GenePix Personal 4100A (Axon, Sunnyvale, CA) and scored by GenePix Pro (Sunnyvale, CA). The spots representing impartial hybridizations (non-uniform intensity) and over hybridizations (too bright) were removed before normalization of the data.

In all, 24 samples were tested that included three independent plants (biological replications) for AGS 2035 and AGS 2060. There were two technical replicates (dye swap) hybridized for each sample.

### Microarray data analysis

Microarray data analysis was implemented in R2.14.2 software (R Development Core Team 2012). The log2 transformed data were normalized within slides using global loess with no background correction or between-slide correction [9]. All normalization was done in the ‘limma’ package [10]. After normalization, the log2 mean value for each probe on each array for each dye was computed; the resulting values were used for further analysis. For differential expression analysis, a mixed model ANOVA using shrinkage estimators with genotype, treatment, genotype by treatment interaction, and dye as fixed effects and biological replicate and slide as random effects was fit in R/maanova [11]. Significance was determined from permutation-based P-values (from 1000 samples) for the genotype and treatment effects and their interaction. P-values were adjusted for the large number of probes tested using the false discovery rate (fdr; [12]). Mean and standard error values were reported in log2 scale after normalization.

### Reverse transcription polymerase chain reaction (RT-PCR)

Seven genes (S1 Table), which showed significant differential expression in AGS 2035 relative to AGS 2060 from the microarray data, were selected for (semi)quantitative RT-PCR analysis. Four varieties, Harrison and AGS 2060 (sensitive) and AGS 2035, USG3120 (resistant) were used for RT-PCR analysis. RNA was extracted from leaf tissues collected at different time points as described earlier. First strand cDNA was synthesized from 1 μg total RNA using iScript cDNA synthesis kit (Bio-rad, Hercules, CA) and diluted four times. One μl of undiluted cDNA was used for semiquantitative RT-PCR and one μl of diluted cDNA (1:4) was used for qRT-PCR following the method described earlier [8]. Wheat elongation factor (EF1A; S1 Table) was used as the internal reference for normalization. The expression of the genes at different time points relative to control (prior to metribuzin application) condition was determined using the 2^^-ddCt^ method as detailed earlier [8].

## Results and discussion

### Varietal response to metribuzin application

**Fig 1:**
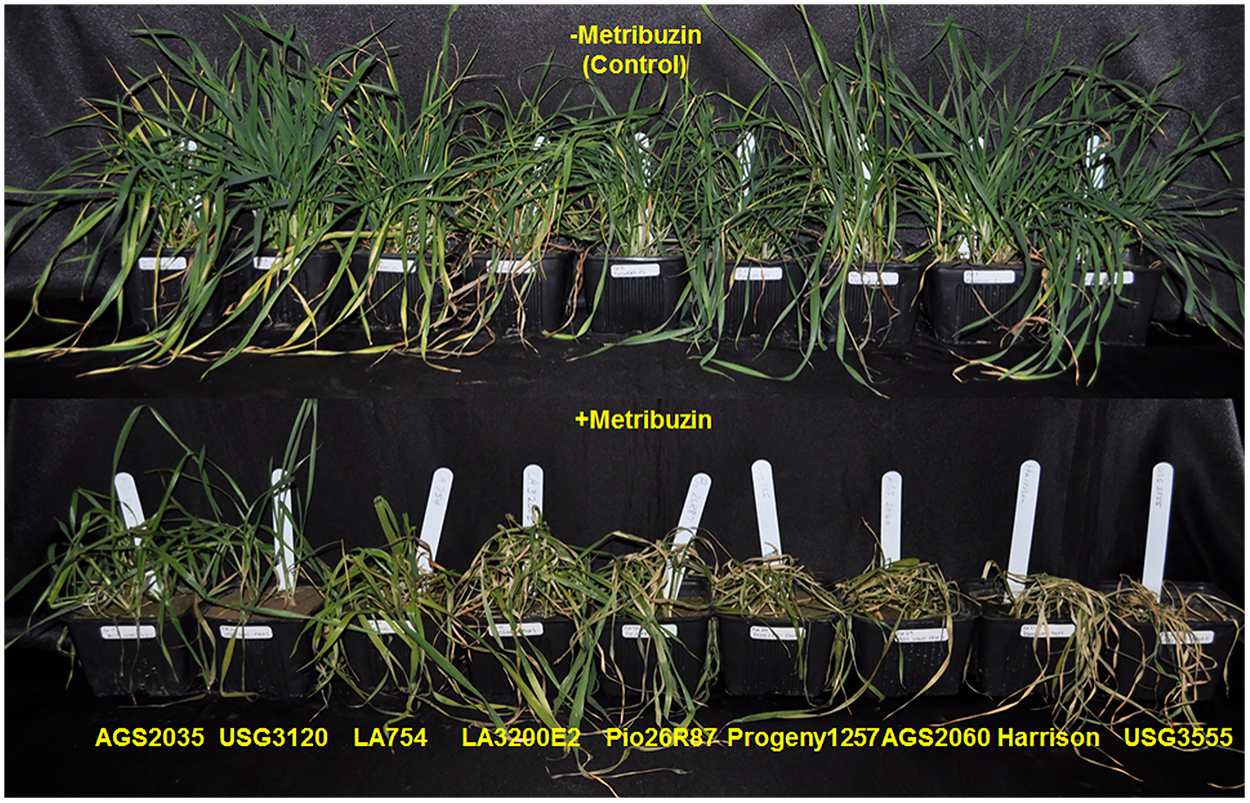
Wheat varieties showing differential response in terms of injury to application of non-selective herbicide, metribuzin. Picture was taken three weeks after herbicide application.

**Fig 2:**
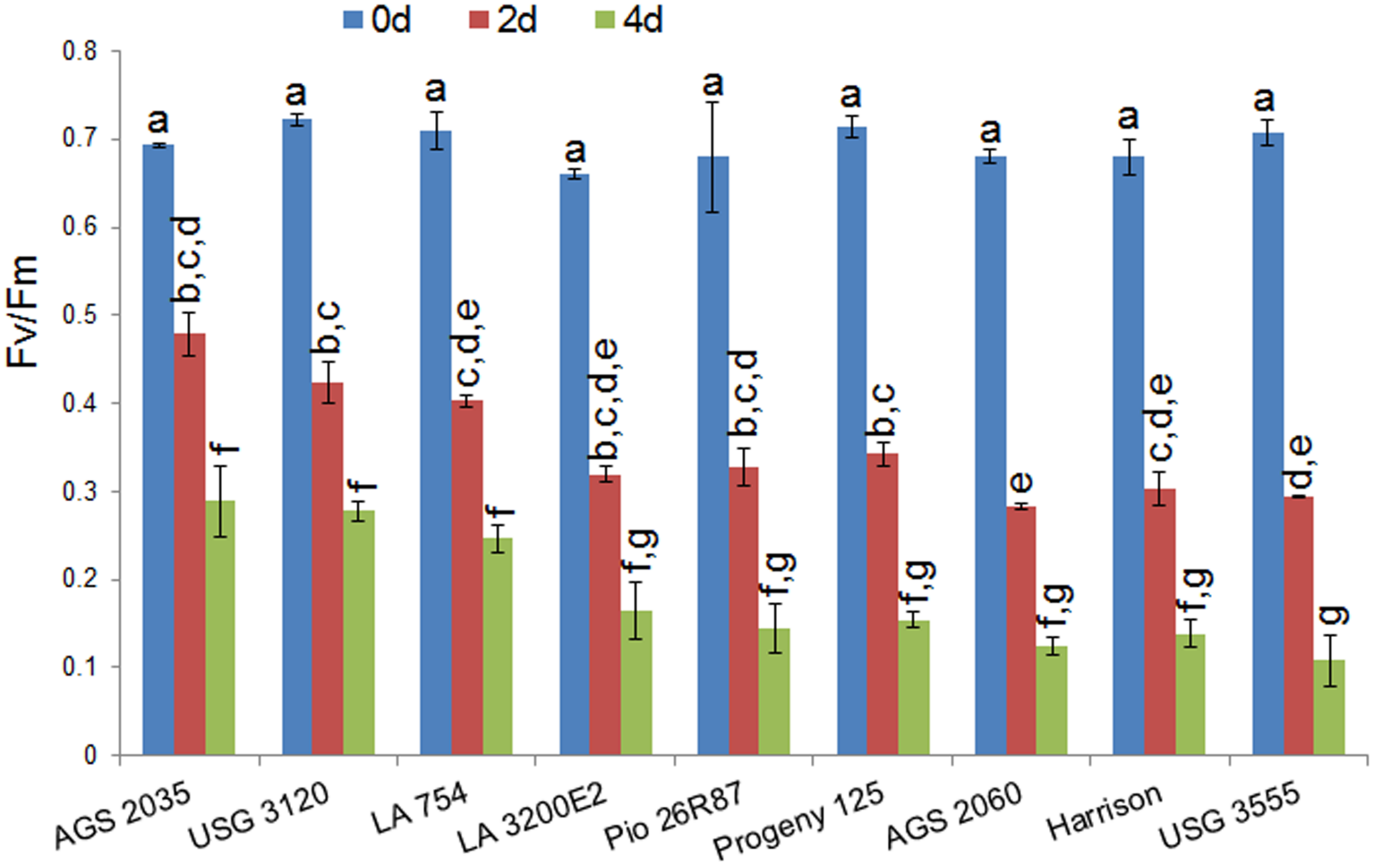
Fv/Fm values indicating photosystem damage of different wheat varieties in response to application of non-selective herbicide, metribuzin. 0d- control (prior to metribuzin application), 2d and 4d- 2 and 4 days after metribuzin application, respectively. Data are presented as mean of values from three plants with the vertical bars representing standard error of means. Bars representing mean values with same alphabets on the top are not significantly different (P<0.05) by Tukey’s HSD test.

The nine varieties showed differential response to metribuzin under controlled conditions inside the growth chamber. Varieties AGS 2035 and USG 3120 were the most tolerant with minimal foliage damage and speedy recovery following a brief slow-down in growth by metribuzin (Fig 1). LA 754 and LA 3200E2 showed moderate tolerance with some damage but were able to recover fairly quickly. Pio 26R87 was moderately sensitive while Progeny 125, AGS 2060, Harrison and USG 3555 were highly sensitive with maximum damage to foliar growth and photosystem II (Fig 2). AGS 2035, LA 754 and USG 3120 had minimum damage to their photosystem with higher Fv/Fm values than the other genotypes. Previous greenhouse and field-based screening also showed that AGS 2035 possessed high levels of resistance to metribuzin whereas AGS 2060 was highly sensitive with severe foliar damage, and tiller death and reduction in growth.

### Gene expression changes in response to metribuzin

Microarray results showed a number of genes being differentially expressed in both AGS 2035 and AGS 2060 varieties. Out of 43,603 probes, 1,429 and 804 transcripts were up/downregulated by metribuzin treatments at P value of 0.05 and 0.01, respectively (GEO Acc. 18532390). One hundred sixty nine and 127 genes were up- and downregulated by at least a 2-fold change in their expression level (S2 Table; S1 Fig). Genes with 5-fold change or above in the expression were considered highly significantly up-/down-regulated and were taken into consideration for further analysis. At 5-fold level, 36 genes were up-regulated and 22 genes were downregulated in response to metribuzin application. Most of these 58 genes were highly upregulated in the resistant variety AGS 2035 in comparison to the susceptible variety AGS 2060.

### Temporal changes in the expression profile of metribuzin-responsive genes

**Fig 3:**
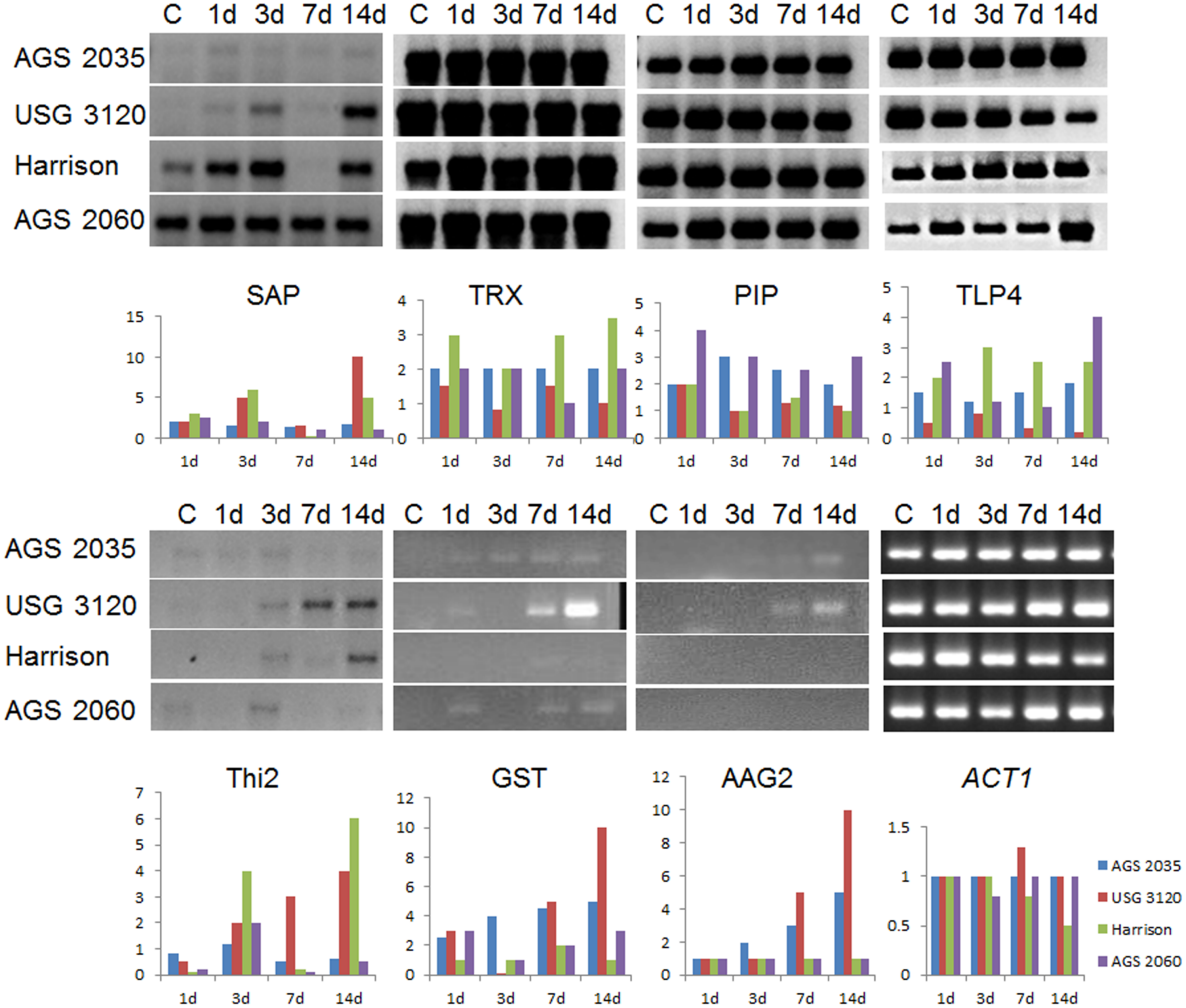
Semiquantitative RT-PCR showing temporal variation in expression of seven genes of wheat in response to metribuzin. SAP, senescence-associate protein; TRX, thioredoxin; PIP, plasmamembrane-intrinsic protein; TLP4, thaumatin-like protein 4; Thi2, thiamine biosynthesis gene 2; GST, galactinol-sucrose galactosyltransferase; AAG2, alkaline alpha glactosidase 2; ACT1, actin 1 (used as internal control). Bar graphs below each gene represent the densitometry-based unreplicated quantitation of the band intensity at different time points relative to control (C). d, day(s) after metribuzin application.

The RT-PCR results (Fig 3, S2 Fig) for seven selected differentially expressed genes (DEGs), which were expressed 2-fold or more in microarray results, showed that almost all of the genes were up-regulated at different time after applying metribuzin as compared to control. Genes that were very highly regulated under metribuzin application were sugar hydrolysis, especially those involved in senescence. Metribuzin resistant varieties USG 3120 and AGS 2035 showed the highest expression level in thioredoxin, a senescence-associated protein, aquaporin PIP1 and a sequence which encodes alkaline alpha-galactosidase or galactinol-sucrose galactosyltransferase.

The gene coding for senescence associated protein (SAP), which was 5.9-fold higher in expression in AGS 2035 than AGS 2060 following metribuzin application (from microarray profiling), showed up-regulation until 3 d after metribuzin application in all varieties. The susceptible varieties had higher mRNA accumulation compared to the resistant varieties. The expression at 7 d was generally low to negligible in all varieties, except AGS 2060. The resistant variety USG 3120 showed highest mRNA content in comparison to control. Basal expression of SAP was generally higher in the susceptible varieties than the resistant varieties. Leaf senescence, a regulated process characterized by chlorophyll degradation, marks the terminal stage of leaf development under normal conditions, but it also occurs under biotic and abiotic stresses [13]. Stress-induced senescence contributes to resource management, recycling and nutrient remobilization for compensating deficiency of photosynthesis and other energy source processes [14]. Metribuzin causes leaf necrosis and senescence by disrupting photosystem II to cause photosynthesis inhibition, metabolite disruption and reactive oxygen species production. The upregulation of SAP may contribute to photosynthate recycling, and nutrient remobilization to help plant cells survive under stress.

Microarray results showed more than 2-fold increase in the expression of the wheat thioredoxin (TRX) gene in resistant variety AGS 2035 as compared to AGS 2060 in response to metribuzin. RT-PCR results showed that all varieties had high basal accumulation of thioredoxin mRNA, under control conditions. Although TRX up-regulation in response to sencor application was clearly evident in Harrison, the content of mRNA was still lower than other varieties especially the resistant ones. Except for the resistant variety AGS 2035, TRX expression was downregulated in three other varieties at 3 d after herbicide application.

Thioredoxin plays a significant role in photosynthesis in plants [15]. In wheat, thioredoxin functions in germination and seedling development by promoting mobilization of primary storage proteins, inactivation of small amylolytic enzyme inhibitors, and activation of calcium-dependent substrate-specific protease [16]. Thioredoxins transcript was shown to increase significantly under oxidative stress conditions in different plants [17]. Herbicide methyl viologen (paraquat) induced an increase in thioredoxin transcript abundance in rice seedlings [18]. Under stress conditions, thioredoxins act as regulators of scavenging mechanisms, such as repairing oxidized proteins and detoxifying lipid hydroperoxides. High basal and sustained expression of TRX in resistant varieties could negate the oxidative bursts due to metribuzin stress, whereas susceptible varieties could tend to produce more thioredoxins in an attempt to protect their cells from more oxidative damage.

Plasma membrane intrinsic protein 1 (PIP1), a member of water channel proteins (aquaporins), was up-regulated by 2.27-fold in response to metribuzin application in the resistant variety, AGS 2035 in comparison with the susceptible variety, AGS 2060, as evident from the microarray data. RT-PCR results showed upregulation of its expression in response to metribuzin in all four varieties. There was no significant difference in PIP1 expression pattern among the varieties although its mRNA content was much higher in USG 3120 and Harrison in comparison with AGS 2035 and AGS 2060.

PIPs play important roles in maintenance of water balance by controlling the transport of water across plant cell membranes, and respond to all abiotic stresses and stress hormones, such as ABA and ethylene [19, 20]. Previous studies indicated that overexpression of PIPs led to rapid water loss and had a negative impact on the growth of the transgenic plants under abiotic stress [21, 22]. Constitutive increase in water transport is harmful in most plant tissues and cells under stress conditions [21, 23]. Most of the herbicides affect photosystem II (PSII) reaction center and block electron transfer leading to production of ROS. Paraquat-induced oxidative stress in cells was found to be indirectly associated with downregulation of aquaporins by ROS leading to reduction of membrane water permeability [24]. ROS are normal products of cellular metabolism, but under stress ROS level increases that damages the plant tissues/cells. The damage to cells biological protein is more noticeable in the susceptible plant, which lacks a powerful ROS scavanging system than tolerant plants. Therefore, susceptible plant cells need to produce more organic molecules to protect the cell life. The present results showed that PIP expression level increased during stress in both tolerant and susceptible varieties but much more in susceptible varieties, which could be due to more damage of ROS in susceptible varieties and consequently increased need of this critical channel protein.

The microarray data showed a 4 to 6 fold upregulation of the gene TLP4 that encoded for thaumatin/osmotin related proteins (TLPs). Semi-quantitative RT-PCR data indicated that the expression level of TLP4 in both resistant varieties AGS 2035 and USG3120 was much greater than susceptible varieties under control condition. The resistant varieties showed a contrasting trend whereas AGS 2035 showed an increased accumulation of TLP4 transcript with time and USG 3120 showed a decline in transcript accumulation in comparison with control. Although there was an upregulation of TLP4 expression in the susceptible varieties with temporal variations, the level of expression was lower compared to the resistant varieties.

TLPs belong to the pathogenesis-related (PR5) protein family, which are often induced under both biotic and abiotic stress conditions, and in response to MeJA and SA treatments [25, 26]. Thaumatin is known to play a role in the plant defense system against pathogens [27, 28]. Thaumatin was upregulated in rice plants overexpressing plant pathogenesis related gene (PR10) that were resistant to both abiotic and biotic stresses [29]. High sequence homology between TLP and osmotin suggests that this low molecular weight protein acts as an osmotin-like protein (OLP), which accumulates at high levels under water stress [30]. This suggests that TLP could be involved in plant’s response to herbicide application as an antioxidant reducing the negative effect of herbicide-induced ROS.

A gene involved in thiamine biosynthesis (Thi2) was downregulated at 1 d after metribuzin application in all varieties except AGS 2035, where the transcript level was unaltered. Although there was an upregulation of its expression at 3 d after herbicide application in all varieties, only resistant variety USG 3120 showed a gradual increase in its mRNA content until 2 weeks after metribuzin application. Thi2 expression was significantly upregulated at 14 d after metribuzin application in the susceptible variety Harrison. In general, Thi2 expression in AGS 2035 was reduced after 3 d of herbicide application but still maintained at a low level unlike AGS 2060, where the expression was not detectable at 7 d and 14 d following metribuzin application.

Thiamine is the active form of vitamin B1 and has a key role in carbohydrate catabolism. Sustained thiamine biosynthesis is required for plant cells to maintain metabolism thereby helping plants to survive under stress [31]. Thiamine accumulation through biosynthesis and exogenous application was shown to help *Arabidopsis* seedlings better tolerate the herbicide, methyl violagen (paraquat) by significantly decreasing the effect of ROS produced by the herbicide effect on protein carboxylation/oxidation [32]. Thiamine plays an important role of an antioxidant by scavenging stress-induced superoxide (O_2_^-.^) and hydroxyl (OH^-.^) radicals. The upregulation of Thi2 in USG 3120 and maintenance of its expression in AGS 2035 suggested that induction of thiamine biosynthesis leading to its accumulation could be one of the important traits of the resistant varieties in response to metribuzin application.

The gene galactinol-sucrose galactosyltransferase (GST) showed a differential expression pattern in response to metribuzin. The gene was induced at 1 d after metribuzin application in all varieties, except the susceptible variety, Harrison. AGS 2035 maintained the expression at a level higher than the control until 1 week, whereas USG 3120, with a downregulation at 3 d, accumulated very high level of transcript at 7 d and 14 d after metribuzin application. Harrison had a very minimal induction of expression at 7 d after metribuzin application, but apparently did not have detectable level of transcript. Two weeks after metribuzin application, AGS 2060, being the susceptible variety, also accumulated GST transcript with a downregulation at 3 d after application.

Galactinol-sucrose galactosyltransferase (GST) belongs to the glycosyltransferases family and plays a significant role in myoinositol and raffinose synthesis pathway. It is also a part of galactose metabolism [33]. Most abiotic stresses induce expression of genes responsible for the raffinose family of oligosaccharides in higher plants, which act as signaling molecules in response to stress conditions, such as pathogen attack and wounding. GST’s function under stress in still unclear, but it catalyzes an exchange reaction between raffinose and sucrose. GST-mediated polymerization of sucrose to the raffinose family of oligosaccharides, which act as compatible solutes, play key roles in plant’s response to most abiotic stresses [34, 35]. The raffinose family of oligosaccharides is also digested by alkaline alphagalactosidases [36].

A glycosyl hydrolase family gene, alkaline alpha-galactosidase 2 (AAG2), was induced during the late stage of metribuzin application in resistant varieties, AGS 2035 and USG 3120. AAG2 was upregulated in resistant varieties AGS 2035 and USG 3120 after one and two weeks of metribuzin application. Susceptible varieties, Harrison and AGS 2060, varieties did not show apparent expression of AAG2 in response to metribuzin. Microarray data analysis showed a 13–17 fold change in expression in response to metribuzin in resistant variety AGS 2035 in comparison with AGS 2060, the susceptible variety.

AAG2 accumlation reprtedly catalyzes galactosyl-saccharide degradation for enhancing carbohydrate utilisation under abiotic stresses [37, 38]. Alkaline alpha-galactosidase can also be induced rapidly under dark, hormones treatment, and biotic stresses. Constitutive, high expression of AAG2 in rice leaves resulted in a severe defect in thylakoid membrane assembly [39, 40]. Metribuzin affects photosynthesis by its action on photosystem II. The resistant varieties must be able to use their stored energy stock under such situations where the plant is unable to provide energy by photosynthesis. AAG2 overexpression in resistant varieties may help plant cells survive by utilizing the carbohydrate supply. The relationship between GST and AAG2 may explain their similar expression patterns in the resistant wheat varieties.

## Conclusion

Metribuzin inhibits a series of reactions involving photosynthesis such as energy conversion, electron transfer and photosynthetic gas exchange, which ultimately can affect the water balance in plant cells. When photosynthesis as a source of energy is inhibited, plant cells need to use their energy storage source to maintain metabolism, via upregulation of enzymes such as alkaline alpha-galactosidase (AAG2) and senescence-associated protein (SAP) involved in these pathways. Alternately, cells of resistant varieties may have promoted some pathways involving enzymes like GST to produce macromolecules and polymers that act compatible solutes to protect cells against herbicide stress. AAG2 and GST were highly expressed in both resistant varieties after metribuzin application to provide a balance between the energy source and protective molecules. On the other hand, although basal expression of SAP was higher in susceptible varieties the expression was induced in resistant varieties only after metribuzin application. Further validation of expression of these three genes (SAP, GST, and AAG) in a diverse germplasm collection will provide a clear understanding of the mode of resistance of wheat to metribuzin as well as to select genes as biomarkers for metribuzin tolerance screening in development of herbicide tolerant wheat. Further, integration of these genes into our ongoing QTL mapping effort will lead to identification of candidate genes within or flanking QTLs for further development of gene-based markers to be used in future for marker-assisted breeding of herbicide tolerance.

## Supporting information

S1 Fig Venn diagram showing significantly (2-fold; P>0.01) up- and downregulated genes in metribuzin resistant (AGS 2035) and sensitive (AGS 2060) wheat varieties. (TIF)

S2 Fig Quantitative RT-PCR showing temporal variation in expression of seven genes of wheat in response to metribuzin. SAP, senescence-associate protein; TRX, thioredoxin; PIP, plasmamembrane-intrinsic protein; TLP4, thaumatin-like protein 4; Thi2, thiamine biosynthesis gene 2; GST, galactinol-sucrose galactosyltransferase; AAG2, alkaline alpha glactosidase 2. EF1A, elongation factor gene was used as reference gene for expression normalization.
(TIF)

S1 Table Primers used for expression analysis of wheat genes differentially expressed by metribuzin reverse transcription polymerase chain reaction. (XLSX)

S2 Table Genes differentially regulated in response to metribuzin application in wheat variety AGS 2035 as compared with AGS 2060. (XLSX)
