# Correction: Genome-wide microarray analysis leads to identification of genes in response to herbicide, metribuzin in wheat leaves

**DOI:** 10.1371/journal.pone.0199564

**Published:** 2018-06-19

**Authors:** Whitney Pilcher, Hana Zandkarimi, Kelly Arceneaux, Stephen Harrison, Niranjan Baisakh

The second author’s name is spelled incorrectly. The correct name is: Hana Zandkarimi. The correct citation is: Pilcher W, Zandkarimi H, Arceneaux K, Harrison S, Baisakh N (2017) Genome-wide microarray analysis leads to identification of genes in response to herbicide, metribuzin in wheat leaves. PLoS ONE 12(12): e0189639. https://doi.org/10.1371/journal.pone.0189639
