# Supplementary figures and images for: Genome-wide microarray analysis leads to identification of genes in response to herbicide, metribuzin in wheat leaves
Source: PLoS One. 2017 Dec 11;12(12):e0189639. doi: 10.1371/journal.pone.0189639 (PMC5724888; doi:10.1371/journal.pone.0189639)

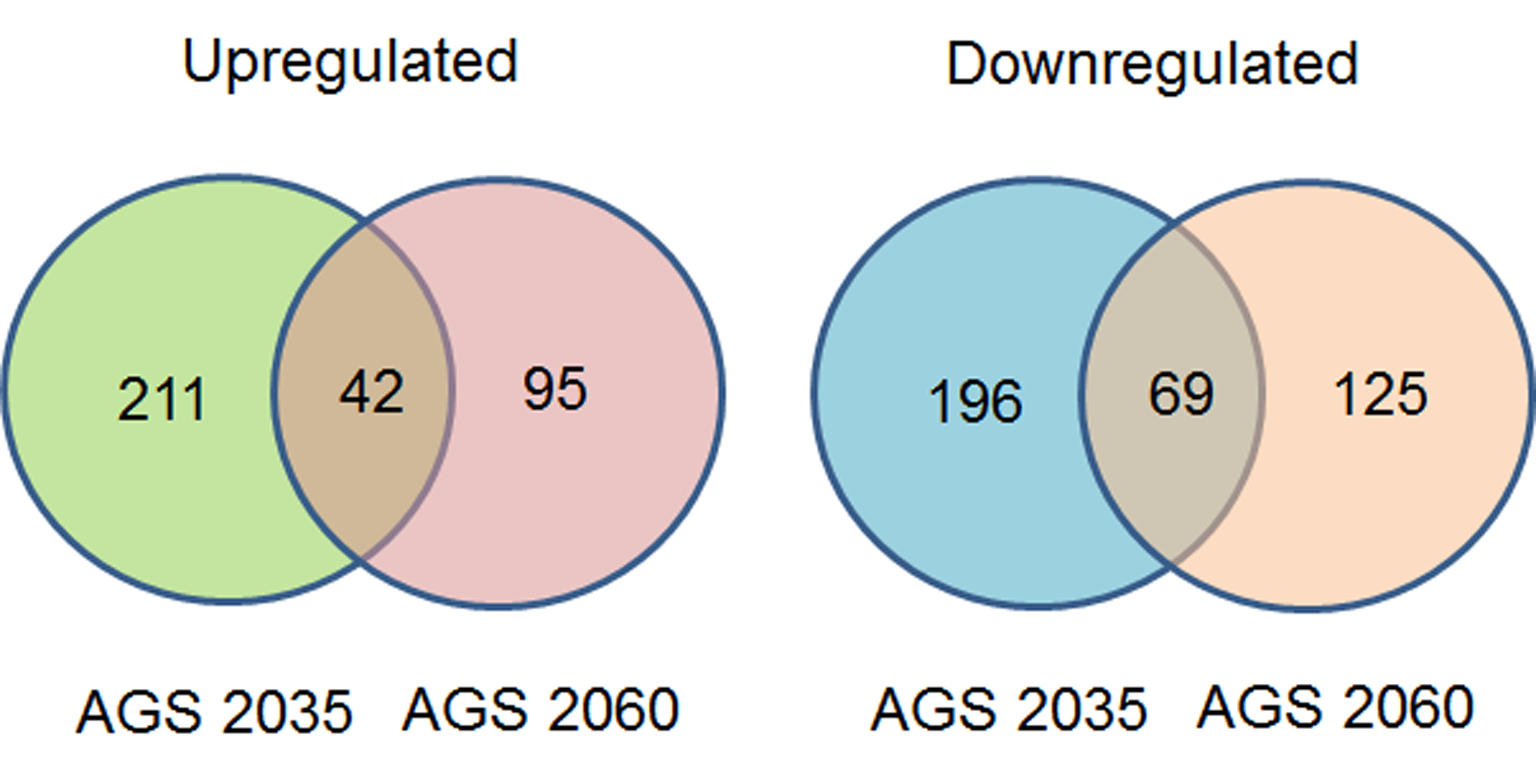

Supplement: S1 Fig — (TIF) [file pone.0189639.s001.tif]

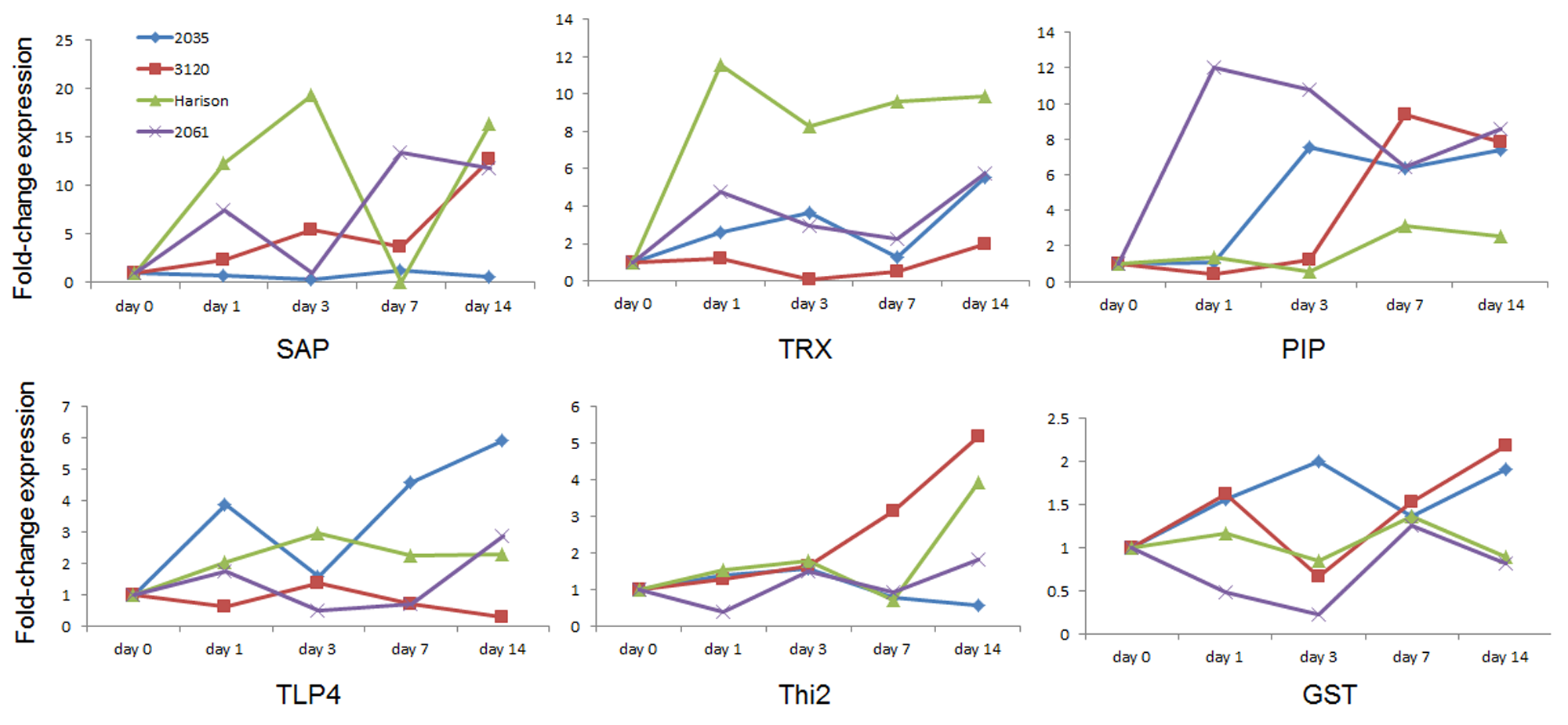

Supplement: S2 Fig — SAP, senescence-associate protein; TRX, thioredoxin; PIP, plasmamembrane-intrinsic protein; TLP4, thaumatin-like protein 4; Thi2, thiamine biosynthesis gene 2; GST, galactinol-sucrose galactosyltransferase; AAG2, alkaline alpha glactosidase 2. EF1A, elongation factor gene was used as reference gene for expression normalization. (TIF) [file pone.0189639.s002.tif]
